# Development and validation of nomogram of peritoneal metastasis in gastric cancer based on simplified clinicopathological features and serum tumor markers

**DOI:** 10.1186/s12885-023-10537-7

**Published:** 2023-01-18

**Authors:** Jia Yang, Hongtao Su, Tao Chen, Xinhua Chen, Hao Chen, Guoxin Li, Jiang Yu

**Affiliations:** 1 Department of General Surgery, Nanfang Hospital, The First School of Clinical Medicine, Southern Medical University, Guangzhou, 510515 Guangdong China; 2 Department of Gastrointestinal Surgery, The Central Hospital of Wuhan, Tongji Medical College, Huazhong University of Science and Technology, Wuhan, 430014 China

**Keywords:** Gastric cancer, Peritoneal metastasis, Nomogram, Clinicopathological features, Serum tumor markers

## Abstract

**Background:**

Peritoneal metastasis (PM) is not uncommon in patients with gastric cancer(GC), which affects clinical treatment decisions, but the relevant examination measures are not efficiently detected. Our goal was to develop a clinical radiomics nomogram to better predict peritoneal metastases.

**Methods:**

A total of 3480 patients from 2 centers were divided into 1 training, 1 internal validation, and 1 external validation cohort(1949 in the internal training set, 704 in the validation set, and 827 in the external validation cohort) with clinicopathologically confirmed GC. We recruited 11 clinical factors, including age, sex, smoking status, tumor size, differentiation, Borrmann type, location, clinical T stage, and serum tumor markers (STMs) comprising carbohydrate antigen 19–9 (CA19-9), carbohydrate antigen 72–4 (CA72-4), and carcinoembryonic antigen (CEA), to develop the radiomics nomogram. For clinical predictive feature selection and the establishment of clinical models, statistical methods of analysis of variance (ANOVA), relief and recursive feature elimination (RFE) and logistic regression analysis were used. To develop combined predictive models, tumor diameter, type, and location, clinical T stage and STMs were finally selected. The discriminatory ability of the nomogram to predict PM was evaluated by the area under the receiver operating characteristic curve(AUC), and decision curve analysis (DCA) was conducted to evaluate the clinical usefulness of the nomogram.

**Results:**

The AUC of the clinical models was 0.762 in the training cohorts, 0.772 in the internal validation cohort, and 0.758 in the external validation cohort. However, when combined with STMs, the AUC was improved to 0.806, 0.839 and 0.801, respectively. DCA showed that the combined nomogram was of good clinical evaluation value to predict PM in GC.

**Conclusions:**

The present study proposed a clinical nomogram with a combination of clinical risk factors and radiomics features that can potentially be applied in the individualized preoperative prediction of PM in GC patients.

**Supplementary Information:**

The online version contains supplementary material available at 10.1186/s12885-023-10537-7.

## Introduction

Gastric cancer is a global health problem, with more than 1 million new cases annually. Although the incidence and mortality of gastric cancer have declined globally in recent years, gastric cancer is still the third leading cause of cancer-related deaths [1, 2]. In recent years, the incidence of gastric cancer has shown a downward trend. However, as a highly invasive and heterogeneous malignant tumor, gastric cancer still maintains a high fatality rate of 75% in most parts of the world, is a major contributor to the global burden of disability-adjusted life years, and remains a global health problem [3–5].

Noninvasive biomarkers can predict the presence of cancer at the early stage and monitor tumor dynamics in real time, as well as predict its prognosis, and have been increasingly used in clinical practice. Serum tumor markers(STMs) have been widely used to diagnose certain populations and monitor the dynamic changes in cancer, for example, CEA, CA19-9 and CA72-4 are three indicators for the diagnosis of gastric cancer, and their elevation is closely related to the occurrence, recurrence and metastasis of gastric cancer [6–8]. In addition, an increasing number of studies have demonstrated that clinicopathological features, tumor size, venous invasion, nodal status, overall stage, tumor type, and distant metastasis are closely related to the prognosis of gastric cancer [9, 10].

Gastric cancer metastasis is the main cause of death in patients with advanced gastric cancer, and the most common sites of metastasis are the liver, peritoneum, lung and bone. At the same time, different sites of metastasis are associated with different survival times, so it is necessary to determine the prognosis of tumor metastasis sites [11–13]. The peritoneum is the most common metastatic site after radical gastrectomy for gastric cancer. Once peritoneal metastasis occurred, the median survival time of patients was only 4 months, while the median survival time of gastric cancer patients without peritoneal metastasis was 14 months [14, 15]. However, peritoneal metastasis is clinically difficult to predict. The accuracy of imaging examinations including computed tomography (CT) and endoscopic ultrasonography (EUS) in diagnosing peritoneal metastasis is not satisfactory [16]. Diagnostic/therapeutic laparoscopy is an effective method for the diagnosis of peritoneal metastasis and is crucial in detecting peritoneal metastasis. However, it is invasive for weak patients who suffer surgical trauma and may place a financial burden on the patient. Therefore, a new model is needed to predict peritoneal metastasis of serous infiltration to formulate effective treatment plans.

In this study, we propose a clinical nomogram method for the preoperative prediction of PM in gastric cancer patients, combining clinical risk factors and radiological characteristics, for individualized preoperative prediction of PM in gastric cancer patients.

## Materials and methods

### Patients and study design

**Fig. 1:**
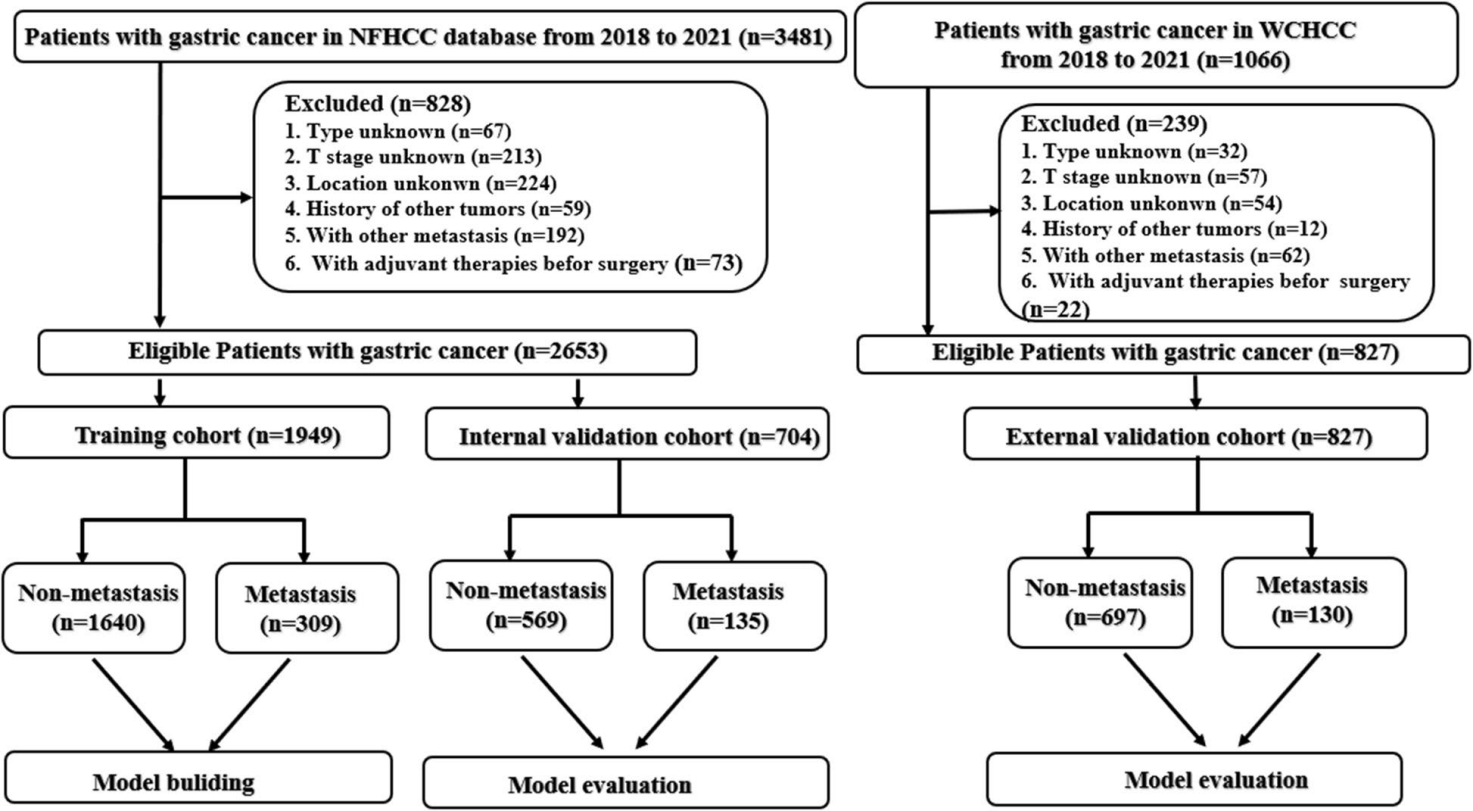
Strategies for selecting patients to be included in the study

From January 1, 2018, to December 31, 2021, a total of 3481 eligible gastric cancer patients from the Nanfang Hospital(NFHCC) dataset and 1066 gastric cancer patients from the Wuhan Central Hospital(WCHCC) cohort were included in the study. A flow diagram of the development and validation of the screening of eligible GC patients is presented in Fig. 1. A total of 3480 GC patients were identified. A total of 1067 patients with the following conditions were excluded from the study. Tumor stage was classified according to the 8th edition of the American Joint Committee on Cancer Staging System. The Ethical Committee and Institutional Review Board of Nanfang Hospital and Wuhan Center Hospital reviewed and approved this study protocol. All procedures performed in studies involving human participants were in accordance with the Helsinki Declaration.

### Data collection

The depth of invasion (clinical T stage) was evaluated by preoperative contrast-enhanced abdominal CT imaging within 4 weeks before surgery, and the Borrmann type was obtained from preoperative diagnostic gastroscopy. Both were classified according to the 8th AJCC tumor classification. Laboratory analysis of STMs was performed via routine blood tests within one week before surgery. The detected STMs included carcinoembryonic antigen (CEA), carbohydrate antigen (CA) 19–9, and CA 72–4. Tumor marker values above these thresholds were considered positive, otherwise, the sample was deemed negative. Peritoneal metastasis was obtained from medical records.

### Construction and validation of the prediction model based on clinicopathological features alone or in combination with STMs

Best subset regression was applied to select the most useful predictive factors from the primary dataset. A predictive score was calculated for each patient via a linear combination of selected features that were weighted by their respective coefficients [17]. To provide clinicians with a quantitative tool to predict the individual probability of PM status, a nomogram based on selected variables was constructed.

### Validation of the prediction model

The calibration of the nomogram was evaluated by the Hosmer–Lemeshow test and displayed in the form of a calibration curve. The accuracy of the predictive ability of the nomogram over time is displayed in the form of an ROC curve, and the discriminatory ability of the nomogram to predict PM in GC is quantitatively expressed by the AUROC. In addition, decision curve analysis (DCA) was conducted to determine the clinical usefulness of the nomogram by quantifying the net benefits at different threshold probabilities.

### Statistical analysis

Statistical analysis was conducted with SAS 9.4 (SAS Institute Inc., Cary, North Carolina, USA) and R3.6.2 (R Foundation for Statistical Computing, Vienna, Austria). The R statistical packages “rms”, “survival”, “Hmisc”,“MASS”, and “timeROC” were used to plot the distribution of risk scores and LNM, build a nomogram, and generate calibration, and time-dependent ROC curves, while “rmda”was used to generate the DCA curves. The categorical variables are expressed as the quantity and percentage, and comparisons were made using the chi-square test or Fisher's exact test where appropriate. All candidate predictors with a *P* < 0.05 in the univariate analysis were included in a multivariate logistic regression model, and all statistical tests were two-sided, with statistical significance set at 0.05.

## Results

### Patients’ clinicopathological characteristics

The clinical characteristics of GC patients in the primary and validation cohorts are presented in Table 1. The detection rate of PM status in the primary cohort was higher than that in the validation cohort (16.7% vs. 15.7%), and there was no significant difference (*P* > 0.05). In both cohorts, PM was found to be significantly associated with age, tumor diameter, tumor location, type, T stage, CEA CA199 and CA 72–4.

**Table 1:** Clinicopathological characteristics of patients in the training, internal validation and external validation cohorts (N(%))

Characteristics		Training cohort		P	Internal validation cohort		P	External validation cohort		P
		Non metastasis	metastasis		Non metastasis	metastasis		Non metastasis	metastasis	
	groups	*N* = 1640	*N* = 309		*N* = 569	*N* = 135		*N* = 697	*N* = 130	
Gender (%)	Male	1105 (67.38)	201 (65.05)	0.4636	379 (66.61)	77 (57.04)	0.0463	476 (68.29)	73 (56.15)	0.0096
	Female	535 (32.62)	108 (34.95)		190 (33.39)	58 (42.96)		221 (31.71)	57 (43.85)	
Age (%)	< 50	438 (26.71)	101 (32.69)	0.037	137 (24.08)	43 (31.85)	0.0798	173 (24.82)	47 (36.15)	0.01
	≥ 50	1202 (73.29)	208 (67.31)		432 (75.92)	92 (68.15)		524 (75.18)	83 (63.85)	
Smoke (%)	No	1218 (74.27)	237 (76.70)	0.4067	439 (77.15)	104 (77.04)	1	522 (74.89)	103 (79.23)	0.3443
	Yes	422 (25.73)	72 (23.30)		130 (22.85)	31 (22.96)		175 (25.11)	27 (20.77)	
Size (%)	< 20 mm	389 (23.72)	13 (4.21)	< 0.0001	151 (26.54)	8 (5.93)	< 0.0001	150 (21.52)	15 (11.54)	0.0126
	≥ 20 mm	1251 (76.28)	296 (95.79)		418 (73.46)	127 (94.07)		547 (78.48)	115 (88.46)	
Differentiation (%)	unknown	926 (56.46)	166 (53.72)	0.1003	335 (58.88)	73 (54.07)	0.4095	405 (58.11)	71 (54.62)	0.2406
	Low	17 (1.04)	1 (0.32)		8 (1.41)	1 (0.74)		13 (1.87)	1 (0.77)	
	Moderate	43 (2.62)	3 (0.97)		16 (2.81)	2 (1.48)		22 (3.16)	1 (0.77)	
	High	654 (39.88)	139 (44.98)		210 (36.91)	59 (43.70)		257 (36.87)	57 (43.85)	
Type (%)	Borrmann I	384 (23.41)	31 (10.03)	< 0.0001	142 (24.96)	18 (13.33)	< 0.0001	153 (21.95)	12 (9.23)	< 0.0001
	Borrmann II	1055 (64.33)	181 (58.58)		355 (62.39)	75 (55.56)		447 (64.13)	84 (64.62)	
	Borrmann III	114 (6.95)	88 (28.48)		47 (8.26)	38 (28.15)		55 (7.89)	32 (24.62)	
	Borrmann IV	87 (5.30)	9 (2.91)		25 (4.39)	4 (2.96)		42 (6.03)	2 (1.54)	
Location (%)	Upper	329 (20.06)	23 (7.44)	< 0.0001	125 (21.97)	11 (8.15)	< 0.0001	144 (20.66)	10 (7.69)	< 0.0001
	Medium	464 (28.29)	142 (45.95)		132 (23.20)	63 (46.67)		174 (24.96)	53 (40.77)	
	Lower	847 (51.65)	144 (46.60)		312 (54.83)	61 (45.19)		379 (54.38)	67 (51.54)	
T stage(%)	unknown	418 (25.49)	104 (33.66)	< 0.0001	146 (25.66)	34 (25.19)	< 0.0001	178 (25.54)	33 (25.38)	< 0.0001
	T1	215 (13.11)	8 (2.59)		86 (15.11)	3 (2.22)		107 (15.35)	2 (1.54)	
	T2	329 (20.06)	23 (7.44)		119 (20.91)	15 (11.11)		126 (18.08)	8 (6.15)	
	T3	678 (41.34)	174 (56.31)		218 (38.31)	83 (61.48)		286 (41.03)	87 (66.92)	
CEA (%)	Negative	1365 (83.23)	206 (66.67)	< 0.0001	456 (80.14)	87 (64.44)	0.0002	569 (81.64)	78 (60.00)	< 0.0001
	Positive	275 (16.77)	103 (33.33)		113 (19.86)	48 (35.56)		128 (18.36)	52 (40.00)	
CA19-9 (%)	Negative	1279 (77.99)	200 (64.72)	< 0.0001	439 (77.15)	74 (54.81)	< 0.0001	547 (78.48)	72 (55.38)	< 0.0001
	Positive	361 (22.01)	109 (35.28)		130 (22.85)	61 (45.19)		150 (21.52)	58 (44.62)	
CA72-4 (%)	Negative	1230 (75.00)	157 (50.81)	< 0.0001	453 (79.61)	62 (45.93)	< 0.0001	524 (75.18)	66 (50.77)	< 0.0001
	Positive	410 (25.00)	152 (49.19)		116 (20.39)	73 (54.07)		173 (24.82)	64 (49.23)	

### The development of a prediction model based on simplified clinicopathological features

**Fig. 2:**
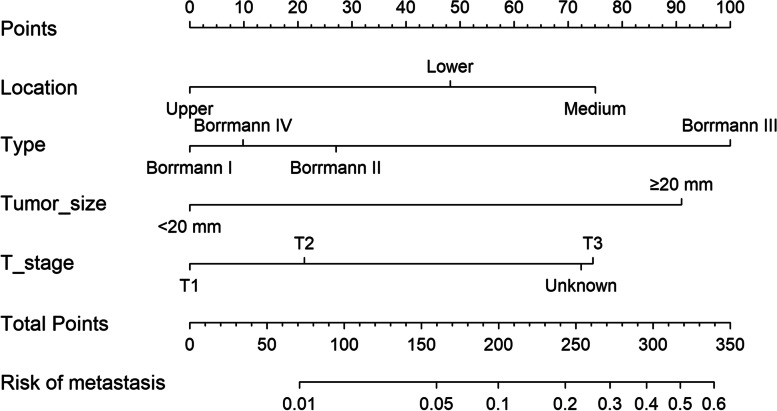
Evaluation of peritoneal metastasis-associated nomograms for gastric cancer patients based on simplified clinicopathological feature

Among the eight simplified clinicopathological features, four variables were selected as the best subset of risk factors to develop the prediction model, including tumor diameter, type, location, and T stage (Table 2). The regression coefficients of multivariate logistic regression models were used to weight each feature in our models. We developed a risk score formula to predict PM status: risk score = -5.919 + 1.695 (if tumor size ≥ 20 mm) + 0.505 (if tumor type Borrmann II; 1.863, if tumor type Borrmann III; 0.183, if tumor type Borrmann IV) + (1.399, if primary location is medium; 0.899, if primary location is lower) + (1.391, if tumor stage is T3). Predicted risk = 1/(1 + e − risk score). The model (Model 1) that incorporated the above predictors was developed and was presented as a nomogram (Fig. 2).

**Table 2:** The logistic regression mode l and mode 2

Variables		Model 1				Model 2			
		β	OR	95%CI	P	β	OR	95%CI	P
Intercept		-5.919			< 0.0001	-6.259			< 0.0001
Size	< 20 mm	reference	1			reference	1		
	≥ 20 mm	1.695	5.447	3.167 – 10.222	< 0.0001	1.539	4.662	2.692 – 8.793	< 0.0001
Type	Borrmann I	reference				reference	1		
	Borrmann II	0.505	1.656	1.109 – 2.545	0.0169	0.488	1.629	1.081 – 2.523	0.0235
	Borrmann III	1.863	6.443	4.013 – 10.561	< 0.0001	1.87	6.486	3.982 – 10.778	< 0.0001
	Borrmann IV	0.183	1.201	0.510 – 2.595	0.6554	0.183	1.200	0.502 – 2.638	0.663
Location	Upper	reference	1			reference	1		
	Medium	1.399	4.049	2.544 – 6.706	< 0.0001	1.475	4.370	2.712 – 7.321	< 0.0001
	Lower	0.899	2.456	1.554 – 4.043	0.0002	0.954	2.597	1.626 – 4.317	0.0001
T stage	T1	reference	1			reference	1		
	T2	0.395	1.484	0.663 – 3.663	0.3591	0.236	1.267	0.558 – 3.156	0.5882
	T3	1.391	4.017	2.020 – 9.184	0.0003	1.205	3.335	1.661 – 7.674	0.0018
	unknown	1.349	3.852	1.907 – 8.898	0.0005	1.273	3.571	1.750 – 8.306	0.0012
CEA					(2.092,7.935)	0.784	2.191	1.602 – 2.989	< 0.0001
CA19-9					(4.792,16.872)	0.223	1.250	0.925 – 1.680	0.1422
CA72-4					(0.851,3.685)	0.866	2.378	1.799 – 3.145	< 0.0001

### Evaluation and External Validation of the PM Prediction Nomogram

**Fig. 3:**
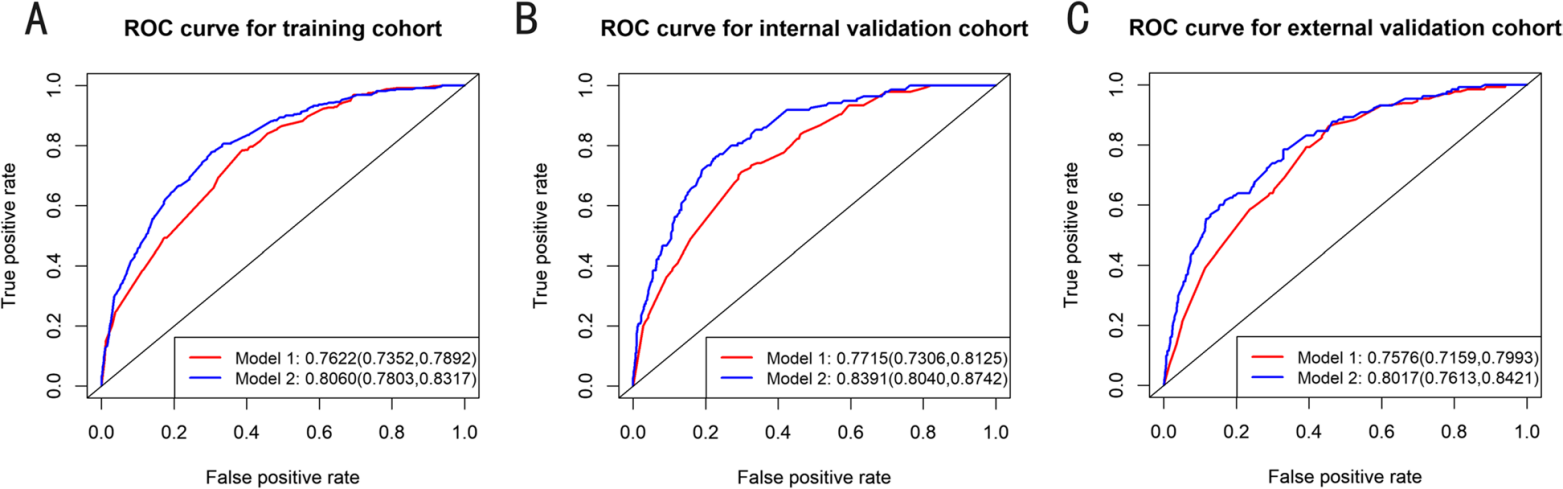
Nomograms of time-dependent receiver operating characteristic (ROC) curves associated with peritoneal metastasis in gastric cancer. **A**, **B** and **C** represent the AUC values of ROC predicted peritoneal metastasis rates of the nomogram in the training, internal validation and external validation cohorts

**Fig. 4:**
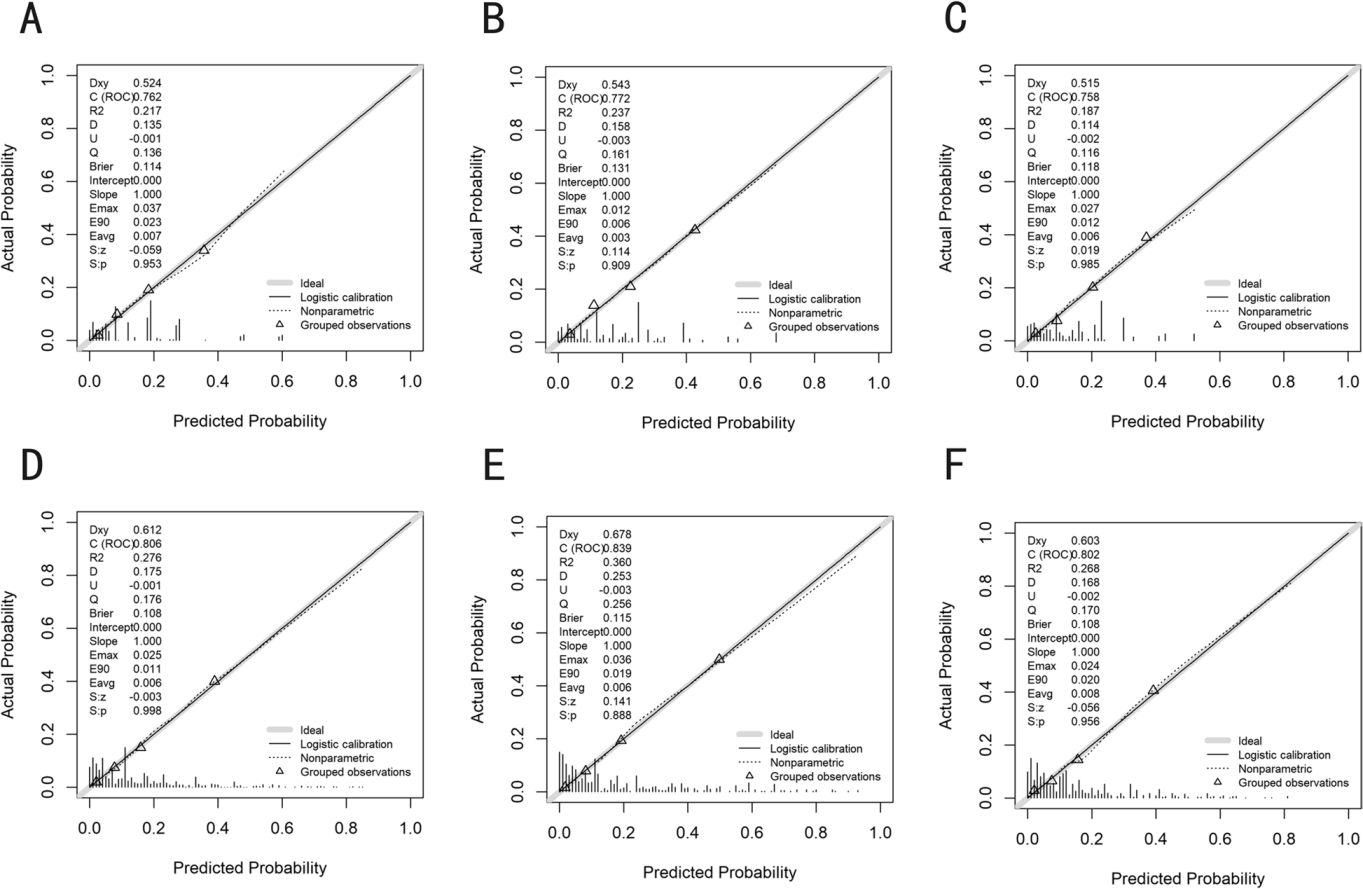
Calibration curves of the prediction models in each cohort associated with peritoneal metastasis in gastric cancer. **A**, **B** and **C** represent the calibration curve for the prediction of peritoneal metastasis in gastric cancer patients in the training, internal validation and external validation cohorts of modle 1; **D**, **E** and **F** represent the calibration curve for the prediction of peritoneal metastasis in gastric cancer patients in the training, internal validation and external validation cohorts of modle 2

The AUC values of the nomogram for the prediction of PM were 0.762 in the training cohort, 0.772 in the internal validation cohort, and 0.758 in the external validation cohort (Fig. 3). The calibration curve of the nomogram for the probability of PM showed good agreement between prediction and observation in the primary cohort (Fig. 4).

### Incremental predictive value of STMs in the above model

To evaluate the additional predictive value of STMs, three STMs, CEA, CA 19–9 and CA 72–4, together with simplified clinicopathological features, were used to develop a PM prediction model. Finally, seven variables were selected as the best subset of risk factors, including, tumor diameter, type, and location, T stage, CEA,CA199 and CA 72–4 (Table 2). The risk score formula of the combined model was as follows: risk score = -6.259 + 1.539 (if tumor size ≥ 20 mm) + 0.488 (if tumor type Borrmann II; 1.87, if tumor type Borrmann III; 0.183, if tumor type Borrmann IV) + (1.475, if primary location is medium; 0.954, if primary location is lower) + (1.205, if tumor Tage is T3) + 0.784 (if CEA is negative) + 0.223 (if CA199 is negative) + 0.866 (if CA 72–4 is positive). Predicted The predicted risk = 1/(1 + e − risk score).

**Fig. 5:**
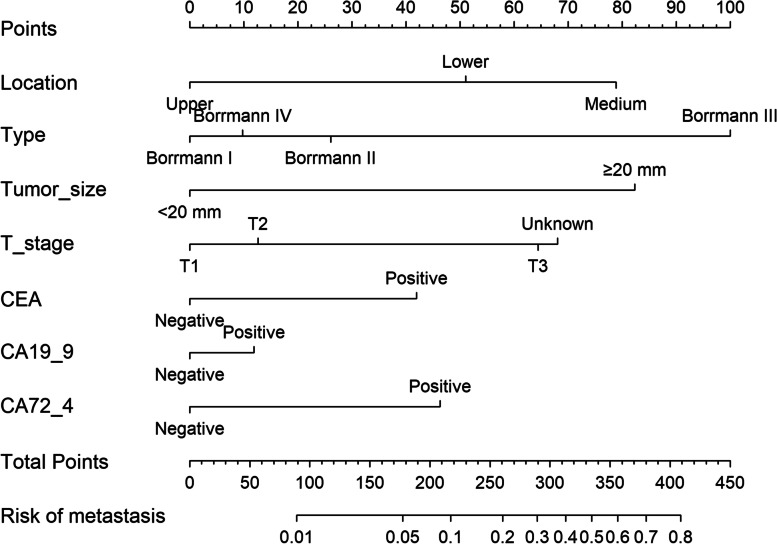
The nomogram integrating simplified clinicopathological features with serum tumor markers

The model (Model 2) that incorporated the above predictors was developed and presented as the nomogram (Fig. 5).

The calibration curve for the probability of PM demonstrated good agreement between prediction and observation in the primary cohort (*P* = 0.998) and validation cohort (*P* = 0.888) (Fig. 4A and Fig. 4B). After the addition of CEA, CA199 and CA 72–4, the discriminatory ability of the pathology-based model was significantly improved in the primary cohort (AUC: 0.806 (95% CI, 0.780 to 0.831) (Fig. 3A), validation cohort (AUC: 0.839 (95% CI, 0.804 to 0.874) (Fig. 3B) and independent validation cohort(AUC: 0.801 (95% CI,0.761 to 0.842), *P* < 0.001) (Fig. 3C).

### Clinical value of the nomogram

**Fig. 6:**
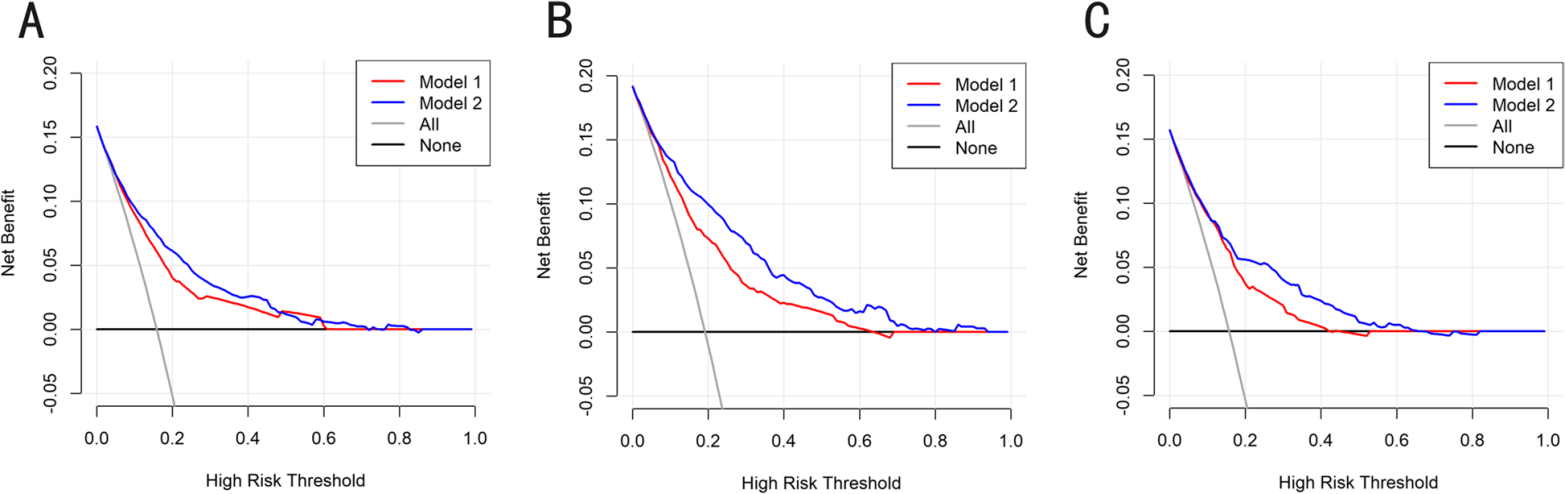
Decision curve analysis of the nomogram for the prediction of peritoneal metastasis in patients with gastric cancer. (**A**) training, (**B**) internal validation and (**C**) external validation cohorts for peritoneal metastasis

DCA is a novel strategy for evaluating alternative predictive treatment methods and has advantages over AUROC in clinical value evaluation. The DCA curves for the developed nomogram in the training, internal validation, and external validation cohorts are presented in Fig. 6.

## Discussion

In our study, we combined clinical risk factors, radiographic characteristics of primary lesions and radiographic characteristics of peripheral blood to establish a prediction model to predict PM in GC patients before surgery. The results of this study suggest that model II has better predictive efficacy in both the training and validation sets, and DCA demonstrates that this nomogram is very useful for predicting PM in GC patients before surgery.

Currrently, the study of PM in GC patients is one of the GC research hotspots and mainly focuses on molecular biology, machine learning and serum markers. However, no standardized and effective PM prediction scheme has been developed because of the individual differences of patients and the particularity of the disease.

Sawaki et al. identified TNNI2 as a candidate for specific overexpression of gastric cancer prone to PM. The high expression of TNNI2 was significantly and specifically correlated with PM [18]. It can be used as an independent risk marker for peritoneal recurrence after radical gastrectomy, One drawback of this study is that the sample size was small, and further validation is needed. Some studies identified corresponding potential targets for PM prediction by comprehensive analysis of the immune spectrum, and clinical and pathological phenotypes based on total exome sequencing (WES) and total transcriptome sequencing (RNAseq) [19]. Nakanishi et al. combined the determination of SYT13 and CEA mRNA levels in peritoneal irrigation fluid to predict peritoneal recurrence of gastric cancer, and the study revealed that peritoneal recurrence risk was highest in patients with positive SYT13 and CEA mRNA levels [19]. Studies also demonstrated that exosomes play a key role in the progression of gastric cancer, through the analysis of malignant abdominal water exosomes in GC patients, exosomes from ascites in GC patients promote EMT signals in GC cells and mouse peritoneal tumor models to promote PM [20]. Kanda et al. conducted a relapse-pattern-specific transcriptomic analysis of 16 patients with stage III gastric cancer, SYT8 was identified as a candidate biomarker for PM specificity, and the high expression level of SYT8 was found to be significantly and specifically correlated with PM [21]. It can be used as an independent prognostic indicator of peritoneal relapse-free survival in patients with stage II/III gastric cancer.

Some studies proposed a prediction model based on collagen characteristics, and demonstrated that high collagen characteristics were significantly correlated with the risk of PM(P < 0.001), which can be conveniently used to independently predict the risk of PM after radical resection of gastric cancer [22]. Jiang et al. trained a deep convolutional neural network to predict occult PM based on preoperative CT images [23]. The results showed that sensitivity and specificity were high in the external validation cohort, and the differential performance of this model was significantly higher than that of conventional clinicopathological factors. Some studies used a random projection algorithm to develop and optimize a machine learning model based on radiomics to predict the advantages of PM in gastric cancer patients. Studies have demonstrated that the precision, sensitivity and specificity of this model are 65.78%, 43.10% and 87.12%, respectively [24]. Zhou et al. used five machine learning methods to establish a PM model, and found that machine learning combining clinical indicators and serum markers could predict PM in gastric cancer [25].

Serum markers also play an important role in predicting PM in gastric cancer. In a retrospective cohort study of patients with advanced gastric cancer, a high preoperative neutrophil/lymphocyte ratio was associated with the presence of PM during staging laparoscopy [26]. Qin et al. found that the serum IgG glycoprotein profile was high in patients with preoperative PM, and IgG glycan was highly correlated with PM, thus,the IgG model may be a reliable prediction scheme [27]. Studies have demontstrated that serum CEA is significantly correlated with poor prognosis of gastric cancer patients, and high serum CA19-9 levels and peritoneal CEA levels are significant predictors of positive peritoneal flushing cytology and peritoneal cancer development, respectively [28]. Similar to this study, our study comprehensively optimized a preoperative PM prediction model for GC patients by combining various serum markers, clinicopathological features and other indicators, to achieve an accurate preoperative prediction for patients without invasive procedures. In summary, it was found that the prediction of PM of GC requires comprehensive prediction and analysis from multiple levels, and there are often some deficiencies in single-level analysis. In our study, we performed a comprehensive analysis of clinically accessible serological indicators and clinicopathological features to achieve a good predictive efficacy for PM Further studies on PM in patients with gastric cancer are needed to improve the estimation of PM.

In our study, the factors we used to establish the prediction nomogram of PM were easily obtained clinically without any invasive procedure or complicated transformation, and with the combined features, the ability of the model to predict PM was improved. Therefore, the model, can be widely used in the clinic. There are limitations of our study. The main limitation is the retrospective nature of the study. Although this study included patients from two different organizations to assess its reproducibility, this research mainly included patients with gastric cancer, and patients in Western countries. therefore, the model's performance in different ethnic groups also needs further evaluation. While the characteristics of conventional clinical and serological markers were analyzed, and through comparison of different models to generate the best model to predict GC patients with PM, the results indicate that further verification is needed.

## Conclusion

In summary, PM is occult for clinical detection in GC patients, and the combined nomogram is an improved model for PM prediction.

## Supplementary Information

**Additional file 1.**

**Additional file 2.**

**Additional file 3.**

## Data Availability

All data generated or analysed during this study are included in this published article and its supplementary information files.
